# Groupwise Image Alignment via Self Quotient Images

**DOI:** 10.3390/s20082325

**Published:** 2020-04-19

**Authors:** Nefeli Lamprinou, Nikolaos Nikolikos, Emmanouil Z. Psarakis

**Affiliations:** Department of Computer Engineering and Informatics, University of Patras, 26504 Patras, Greece; lamprinou@ceid.upatras.gr (N.L.); nikolikos@ceid.upatras.gr (N.N.)

**Keywords:** groupwise registration, congealign, image alignment, medical imaging, photometrically distorted image alignment, partially occluded image alignment, multimodal alignment, self quotient image

## Abstract

Compared with pairwise registration, the groupwise one is capable of handling a large-scale population of images simultaneously in an unbiased way. In this work we improve upon the state-of-the-art pixel-level, Least-Squares (LS)-based groupwise image registration methods. Specifically, the registration technique is properly adapted by the use of Self Quotient Images (SQI) in order to become capable for solving the groupwise registration of photometrically distorted, partially occluded as well as unimodal and multimodal images. Moreover, the proposed groupwise technique is linear to the cardinality of the image set and thus it can be used for the successful solution of the problem on large image sets with low complexity. From the application of the proposed technique on a series of experiments for the groupwise registration of photometrically and geometrically distorted, partially occluded faces as well as unimodal and multimodal magnetic resonance image sets and its comparison with the Lucas–Kanade Entropy (LKE) algorithm, the obtained results look very promising, in terms of alignment quality, using as figures of merit the mean Peak Signal to Noise Ratio ($m_{PSNR}$) and mean Structural Similarity ($m_{SSIM}$), and computational cost.

## 1. Introduction

Groupwise image alignment/registration or congealign is a joint alignment process that handles a large scale of images simultaneously, in contrast to pairwise alignment/registration. The goal is to align all images with one another in an unbiased way, no specific image should introduce a registration bias. A good congealing algorithm can be used as preprocessing to notably improve the performance of other vision tasks within different research areas such as medical, satellite and aerial image registration [1,2,3].

Congealign algorithms tend to utilize one image at a time as the held out image and the rest as the stack, that keep changing while a dissimilarity/similarity function is iteratively minimized/maximized. This is done by estimating warp updates for each image that best align them with the stack. Methods based on the aforementioned idea include groupwise methods with entropy-based cost functions as well as LS-based cost functions. In [4] entropy-based congealign was introduced, by defining a method that minimizes the joint entropy across pixel stacks distributions. In [5], entropy-based congealing was adapted to handle large sets of gray-scale valued, 3D medical images. In [6] the need for ad hoc regularization of the calculated transformations over an iteration was removed. In [7] and [8] Least Square (LS)-based methods were introduced, using sum-of-squared-differences (SSD)-based objective functions. Their efficiency compared to entropy-based techniques was demonstrated, regarding both the convergence rate and accuracy. In the LS framework, given a held out image and a stack and then calculating the warp update to align the former to the latter (forward approach) results in lower computational costs but also lower accuracy. In contrast, calculating the updates to align the stack to the held out image (inverse approach) [7] results in better accuracy but also higher complexity, due to nested loops, a fact that makes its use prohibitive for large image sets. Moreover, the problem of outliers can be handled by ideas such as the one presented in [8]. In [9] the impact of utilizing features with LS-based congealign was investigated. In [10] a Gradient Correlation Coefficient objective function was introduced as an improvement upon the LS-based framework, given that the maximization problem introduced has an LS-based problem that is equivalent. In [11] an error function-based on Mutual Information that copes with possible variations in appearance between similar objects of the same class is defined. In addition, in [12] and [13] extended entropy-based congealing for the usage on the real-world complex images is proposed while in [2] a variational Bayesian approach for ensemble registration is presented. Recently, in [14,15] groupwise registration techniques tailored for the registration of quantitative MRI datasets were presented.

In recent years, deep learning methods, based on deep architectures of neural networks (DNN’s), have been the state of the art in solving various computer vision problems. Classical problems such as human, head and hand pose estimation, object detection, image segmentation, image understanding, object tracking, pattern recognition have been successfully solved by using such deep networks [16,17,18,19]. Pairwise image alignment has also attracted the attention of the scientific community, and several DNN schemes have been proposed for its successful and computationally cheaper [20] solution. Supervised deep autoencoder-based schemes for fast predictive image registration by adopting a patch-wise prediction strategy for large deformation of the diffeomorphic metric mapping model, unsupervised deep learning framework based on ConvNets for affine and deformable image registration, reinforcement learning deep similarity-based methods and Generative Adversarial Networks (GAN) have been proposed [21]. Lately, DNN solutions have been proposed for the groupwise alignment problem [22,23], showing promising results.

The proposed groupwise method shows the accuracy of the inverse logic, but still maintains a linear to the size of the ensemble computational cost. The use of the self quotient images instead of the original ones permits the successful solution of the multimodal image alignment problem. In addition, by using feature descriptors instead of raw intensity values, as representations for the images, background variations can be dealt with.

The remainder of this paper is organized as follows: in Section 2, we formulate the image congealing problem as a parametric nonlinear optimization problem and the main related issues with it, are examined. In particular, the need of the simultaneous minimization with respect to the parameter vectors of the geometric transformations as well as the unknown “mean” image of a least-squares-based cost function in each iteration of the optimization process is indicated and an alternate approach is proposed in order to achieve a computationally efficient but sub-optimal solution of the problem. In Section 3, the multimodal image alignment problem is presented and the use of self quotient images (SQI) for solving the multimodal alignment problem is proposed. The benefits of using these kinds of images to solve the image alignment problem are analyzed. In Section 4, the results we have obtained from the application of the proposed method in a number of experiments we have conducted are presented. Moreover, the performance of the proposed method is compared against the Lucas–Kanade Entropy method [24], a technique that outperforms well-known image alignment methods [10], and its superiority is shown. Finally, Section 5 concludes our paper.

## 2. Problem Formulation

### 2.1. Preliminaries

Adopting the notation used in [25], we consider the following sets of images:

(1) $$\begin{array}{ccc} S_{i} & = & {\left\{i_{n}\right\}}_{n=1}^{N} \end{array}$$

(2) $$\begin{array}{ccc} S_{i_{w}}\left(P_{N}\right) & = & {\left\{i_{w}\left(p_{n}\right)\right\}}_{n=1}^{N}. \end{array}$$

Set $S_{i}$ contains a group of *N* similar in shape and aligned images, with i denoting the column-wise of length $N_{x}N_{y}$ vectorized version of size $N_{x}\times N_{y}$ image *I*, while set $S_{i_{w}}\left(P_{N}\right)$ contains the geometrically distorted vectorized images of set $S_{i}$ in (1). This latter set depends on the set

(3) $$P_{N}={\left\{p_{n}\right\}}_{n=1}^{N},$$

containing *N* warp parameter vectors used in warping transformation $w(\;.\;;p_{n})$ which is parameterized by the vector $p_{n}\;\in \;R^{M}$, that deforms the support of image $i_{n}$, of set $S_{i}$ and maps its values onto the corresponding image $i_{w}\left(p_{n}\right)$ of set $S_{i_{w}}\left(P_{N}\right)$. In this paper to model the warping process we use the class of affine transformations with $p_{n}\;\in \;R^{6}$.

Then, groupwise registration, or congealing [1] can be defined as the minimization problem of a misalignment function, let us denote it by $C(P_{N})$, which is defined over the set $S_{i_{w}}\left(P_{N}\right)$. Solving this problem is not an easy task and its complexity depends on several factors, such as the size of the ensemble, the size of images and the strongness of the geometric distortions, to name a few, and in most cases its solution results in a highly nonlinear and computationally demanding procedure. This is basically because the goal of estimating the set $P_{N}$ of the unknown parameters should be achieved by defining a misalignment function $C(P_{N})$ over the entire ensemble of images. Such a function, which is known as the Cumulative Squared Misalignment Error (CSME):

(4) $$C\left(P_{N}\right)=\sum_{n=1}^{N}\epsilon \left(p_{n}\right),$$

where

(5) $$\epsilon \left(p_{n}\right)=\sum_{m=1,m\neq n}^{N}\left|\right|i_{w}\left(p_{n}\right)-i_{w}\left(p_{m}\right){\left|\right|}_{2}^{2},$$

was proposed in [7]. However, this total cost function is difficult optimize directly [26] and the minimization of the individual cost function $\epsilon (p_{n})$ for each geometrically distorted image $i_{w}\left(p_{m}\right)$, that was proposed, demands the solution of $O(N^{2})$ pairwise alignment problems.

Instead of the CSME defined in Equation (4), in [25] the following total mean misalignment function:

(6) $$C_{0}\left(P_{N};{\bar{i}}^{★}\right)=\frac{1}{N}\sum_{n=1}^{N}\left|\right|{\bar{i}}^{★}-i_{w}\left(p_{n}\right){\left|\right|}_{2}^{2}$$

was proposed, with ${\bar{i}}^{★}$ denoting the “mean” image of set $S_{i}$, that is:

(7) $${\bar{i}}^{★}=\frac{1}{N}\sum_{n=1}^{N}i_{n}$$

that constitutes the most representative image of the above mentioned set.

Assuming that the “mean” image is known, it is clear that the above misalignment function, although non-linear with respect to each member of the set of warp parameters defined in (3), is separable and demands the solution of O(N) pairwise alignment problems. In such a case, for each one of the cost functions involved in (6) its minimization requires nonlinear optimization techniques either by using direct search or by following gradient-based approaches.

It is a common strategy in iterative techniques the original minimization problem to be replaced by a sequence of secondary ones. Each secondary problem relies on the outcome of its previous one, thus generating a chain of parameter estimates which hopefully converges to the desired optimal solution of the original problem. Adopting an additive update rule for the parameters vector, that is $p_{n}\left(k\right)=p_{n}\left(k-1\right)+\Delta p_{n}\left(k\right)$, where $\Delta p_{n}\left(k\right)$ denotes a vector of perturbations, their optimal values result from the solution of the following optimization problem:

(8) $$\min_{\Delta P_{N}\left(k\right)}C_{0}\left(\Delta P_{N}\left(k\right);\;{\bar{i}}^{★}\right)$$

with the cost function $C_{0}\left(\Delta P_{N}\left(k\right);\;{\bar{i}}^{★}\right)$ defined as follows

(9) $$C_{0}\left(\Delta P_{N}\left(k\right);\;{\bar{i}}^{★}\right)=\frac{1}{N}\sum_{n=1}^{N}\left|\right|{\bar{i}}^{★}-i_{w}\left(p_{n}\left(k-1\right)+\Delta p_{n}\left(k\right)\right){\left|\right|}_{2}^{2}$$

and the set $\Delta P_{N}\left(k\right)$ contains the *N* perturbations of the warp parameter vectors at the *k*-th iteration of the minimization process, that is:

(10) $$\Delta P_{N}\left(k\right)={\left\{\Delta p_{n}\left(k\right)\right\}}_{n=1}^{N}.$$

The optimal solution of the optimization problem (8) is given by:

(11) $$\Delta p_{n}\left(k\right)=A_{w}\left(p_{n}\left(k-1\right)\right)\left({\bar{i}}^{★}-i_{w}\left(p_{n}\left(k-1\right)\right)\right)$$

where

(12) $$A_{w}\left(p_{n}\left(k-1\right)\right)={\left(G_{w}{\left(p_{n}\left(k-1\right)\right)}^{T}G_{w}\left(p_{n}\left(k-1\right)\right)\right)}^{-1}G_{w}{\left(p_{n}\left(k-1\right)\right)}^{T}$$

is the $M\times N_{x}N_{y}$ pseudo inverse of the Jacobian matrix $G_{w}\left(p_{n}\left(k-1\right)\right)$ evaluated at $p_{n}\left(k-1\right)$ [27].

Note however, that since the “mean” image ${\bar{i}}^{★}$ is unknown, the optimal values of the perturbations in (11) can not be computed. For avoiding this obstacle a strategy based on the use of a particle system and imposing its center of mass to be motionless during the optimization process was proposed in [25]. In the next paragraph, we propose the solution of the above-mentioned problem formulating a different optimization problem.

### 2.2. The Proposed Solution

To this end, let us define the following sequence of vectorized images:

(13) $$I_{c}={\left\{i\left(k\right)\right\}}_{k=1}^{\infty },$$

with the following two properties:

- $P_{1}$: The *k*-th member of the sequence, is an approximation of the “mean” image in the *k*-th iteration of the minimization process
- $P_{2}$: The limit of the sequence we would like to be the unknown “mean” image, that is:
  (14) $$\lim_{k\to \infty }i\left(k\right)={\bar{i}}^{★}.$$

Then, we can redefine the secondary cost function (9) as follows:

(15) $$C_{1}\left(\Delta P_{N}\left(k\right),\;i\left(k\right)\right)=\frac{1}{N}\sum_{n=1}^{N}||i\left(k\right)-i_{w}\left(p_{n}\left(k-1\right)+\Delta p_{n}\left(k\right)\right){\left|\right|}_{2}^{2}$$

and our goal now is its double minimization; namely, with respect to parameter’s set $\Delta P_{N}\left(k\right)$ as well as the *k*-th “mean” image i(k).

Solving the above optimization problem does not constitute a very difficult task. However, the computation of its optimal solution is expensive.

We can avoid this obstacle by defining a cost function that can be minimized with respect to the “mean” image and in combination, in an alternating way, with the minimization of the cost function $C_{0}\left(\Delta P_{N}\left(k\right);\;{\bar{i}}^{★}\right)$ (9), permits at each iteration a computationally cheap, but not necessarily equivalent, solution of the desired problem.

To this end, let us define the following cost function:

(16) $$C_{2}\left(i\left(k\right);\;P_{N}\left(k-1\right)\right)=\frac{1}{N}\sum_{n=1}^{N}||i\left(k\right)-i_{w}\left(p_{n}\left(k-1\right)\right){\left|\right|}_{2}^{2},$$

where the parameter’s set $P_{N}\left(k-1\right)$ is known and we would like to minimize with respect to the *k*-th approximation of the “mean” image i(k). It is clear that the new cost function is strongly related to both above defined cost functions $C_{0}\left(P_{N}\left(k-1\right);\;i\left(k\right)\right)$ and $C_{1}\left(\Delta P_{N}\left(k\right),\;i\left(k\right)\right)$ in (6) and (15) respectively.

Minimization of the cost function $C_{2}\left(i\left(k\right);\;P_{N}\left(k-1\right)\right)$ with respect to the “mean” image i(k), results in the following optimal solution:

(17) $$i\left(k\right)\equiv i_{w}\left(k\right)=\frac{1}{N}\sum_{n=1}^{N}i_{w}\left(p_{n}\left(k-1\right)\right),$$

which, actually, is the average of the warped vectorized images and its computation is cheap. An outline of the proposed algorithm shown in Algorithm 1.
**Algorithm 1:** Outline of the Proposed LS-Groupwise Algorithm
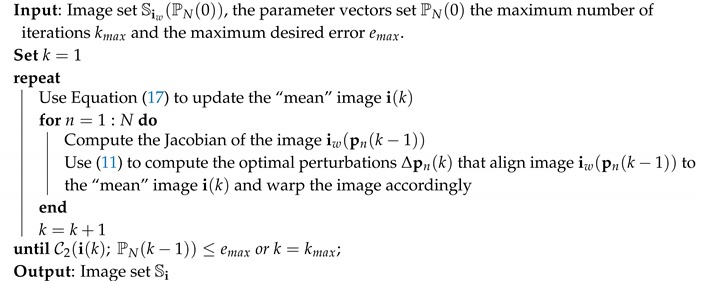


**Figure 1:**
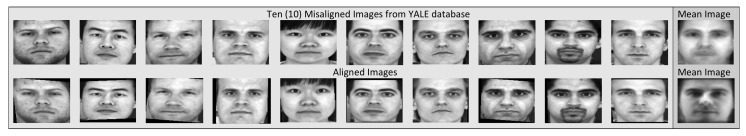
Ten strongly geometrically deformed images from Yale database as well as their mean image (first row). Their aligned counterparts with their corresponding mean (second row) after their groupwise alignment by the proposed algorithm.

Before we proceed in presenting the image registration problem in the case of multi modal images, let us apply the above-mentioned algorithm in the groupwise alignment problem of the ten strongly geometrically deformed images from the Yale database [28] shown in the first row of Figure 1. The strongness of the geometric deformations is evident in their mean image which is shown at the end of first row of this figure. In the second row of this figure we can see the results we have obtained after 20 iterations of the proposed algorithm. The successful alignment of the image set can be validated by looking at their mean image which is clearly enhanced, compared to the original mean image before the application of the proposed groupwise alignment technique.

## 3. Registration of Multimodal Images

In this section, we are going to examine the registration problem of images of different modalities. Such problems appear when we want to register photometrically deformed and/or occluded images as well as MR Images of different modalities, to name a few. We are going to present examples of photometrically deformed and partially occluded images that we would like to align. Then, we present the existing different modalities of MR images. Finally, an edge-preserving filtering scheme, originally proposed in [29], used for their preprocessing will be shortly explained.

### 3.1. Photometrically—Distorted Images

**Figure 2:**
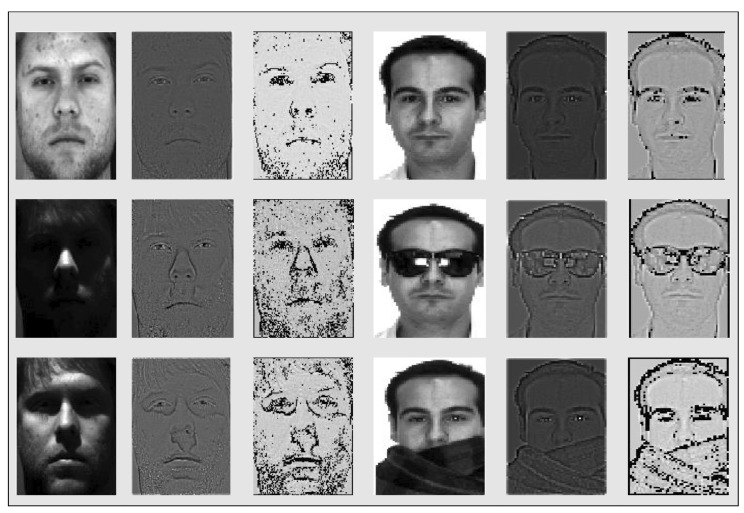
Photometrically distorted images from Yale database (first column) and their Self Quotient Images (SQI) counterparts before (second column) and after thresholding (third column). Images with occluded areas from Yale database (fourth column) and their SQI counterparts before (fifth column) and after thresholding (sixth column).

In this paragraph, we focus on the alignment of photometrically distorted and occluded images, that constitutes a well-known and difficult problem [30,31]. In these kinds of alignment problems, there is a large number of outliers, thus not all pixels must be used during the optimization. In the first and fourth column of Figure 2, three photometrically distorted images from the Yale database [28] and an equal number of images with occluded areas from the AR database [32] are shown. Clearly, these images have totally different intensity distributions.

### 3.2. Multimodal MR Images

**Figure 3:**
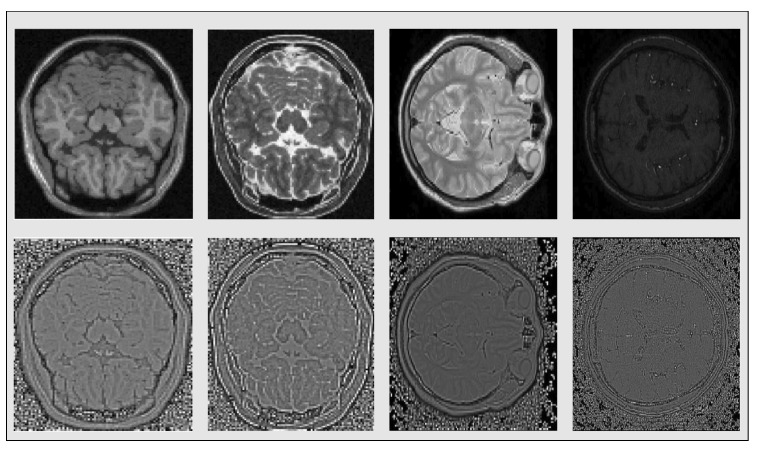
Original T1, T2, Proton Density (PD) and magnetic resonance angiography (MRA) images respectively (first row) and their SQI’s counterparts after thresholding (second row).

In MR Images the contrast depends on the magnetic properties and the number of hydrogen nuclei existing in the area being imaged. Common type MR Images include T1 and T2-weighted resulting from different timing radiofrequency pulse sequences, Proton Density (PD) that display the number of nuclei in the area and magnetic resonance angiography (MRA) that highlights movement in the body’s blood vessels, among others. These different types are presented in Figure 3. Examining different types of medical images, such as different types MRIs, CT images etc, that have totally different intensity distributions, can often be presented as a problem of strong photometric distortions, so we are going to address it as such in the next subsection.

### 3.3. Self Quotient Images

Considering images such as presented above, the use of intensity-based techniques for aligning, either pairwise or groupwise, is not a good choice for the solution of the problem. In order to be able to use such area-based techniques, the preprocessing of the images with a known edge-preserving filter [33], was proposed in [29], which is briefly presented in this section. The SQI is defined as:

(18) $$Q\left(x\right)=\frac{I(x)}{I_{\sigma }\left(x\right)}\;,\forall x\;\mathrm{in}\;\mathrm{the}\;\mathrm{support}\;\mathrm{of}\;I\left(x\right),$$

where $I_{\sigma }\left(x\right)$ is a smoothed version of the image I(x) resulting from its convolution with the isotropic Gaussian kernel $G_{\sigma }\left(x\right)$, with the subscript denoting its standard deviation.

Note that the deviation of the Gaussian kernel controls the width of the edges in the image defined in Equation (18). To address the noise as well as the outliers problem from which SQI suffers from we use a hard thresholding procedure. To this end, let $\sigma_{Q}$ be the standard deviation of the vectorized counterpart of SQ Image Q(x),withxin the support ofQ(x). Then, we can define the following threshold:

(19) $$T_{\mu }=\mu \;\sigma_{Q}$$

with 0<μ<1 a data-dependent parameter that can be used to have additional control over the value of threshold $T_{\mu }$ and use it for “denoising” purposes. In all the experiments we conducted we set that parameter equal to 0.5. The SQIs resulting from the application of the above-mentioned procedure on the photometrically distorted images and the four different contrast type MRI slices, are shown in Figure 2 and Figure 3 respectively. Having filtered out the strong photometric distortions, we can use the above mentioned area-based technique to solve the groupwise alignment problem.

## 4. Experiments

In this section, we are going to present our results. In order to demonstrate the performance of the proposed technique, we conducted three experiments. To test the effectiveness in highly deformed sets of images we applied warps to the original images using the framework presented in [27] was used, with the distortion parameter $\sigma^{2}$ taking values in the interval [1, 10], with the values 1 and 10 corresponding to the smallest and strongest geometric distortions respectively.

Specifically, we are going to evaluate the performance of the proposed against Lucas–Kanade Entropy [24]-based technique, a technique that outperforms other well-known alignment methods such as least square congealing [7], data-driven image models through continuous joint alignment [4], and robust alignment by sparse and low-rank decomposition for linearly correlated images [34]. Gradient Correlation Coefficient-based techniques [10], as it was already mentioned, are appropriate for the small size of image sets (N≤100). Its computational cost is prohibitive for sets of greater cardinalities [10] and special strategies must be followed for solving efficiently the groupwise alignment problem.

**Figures of Merit:** Since there are not ground truth images, in order measure performance we are going to use as figures of merit the “mean” Peak Signal to Noise Ratio ($m_{PSNR}$) and the “mean” Structural Similarity ($m_{SSIM}$). To this end, for the computation of the “mean” PSNR we compute the PSNR of each image $i_{w}\left(p_{n}\left(k_{max}\right)\right),\;n=1,2,\cdots ,N$ of the ensemble with respect to the “mean” $N_{x}\times N_{y}$ image $i(k_{max})$ obtained after $k_{max}$ iterations of the algorithms, i.e.,

(20) $$PSNR\left(n\right)=10\log\left(\frac{\max{\left\{i\left(k_{max}\right)\right\}}^{2}}{MSE(n)}\right),$$

where the MSE(n) is the mean squared error between the “mean” image $i(k_{max})$ and $i_{w}\left(p_{n}\left(k_{max}\right)\right)$, that is:

(21) $$MSE\left(n\right)=\frac{1}{N_{x}N_{y}}||i\left(k_{max}\right)-i_{w}\left(p_{n}\left(k_{max}\right)\right){\left|\right|}_{2}^{2},$$

and take their mean value:

(22) $$m_{PSNR}=\frac{1}{N}\sum_{n=1}^{N}PSNR\left(n\right).$$

Similarly, for the computation of the ”mean” SSIM we compute the SSIM of each image with the “mean” one, i.e.,

(23) $$SSIM\left(n\right)=\frac{\left(2\mu_{0}\mu_{n}+c_{1}\right)\left(2\sigma_{0n}+c_{2}\right)}{\left(\mu_{0}^{2}+\mu_{n}^{2}+c_{1}\right)\left(\sigma_{0}^{2}+\sigma_{n}^{2}+c_{2}\right)}.$$

where $\mu_{0}$, $\mu_{n}$, are the mean values of the “mean” image $i(k_{max})$ and the warped image $i_{w}\left(p_{n}\left(k_{max}\right)\right)$ respectively, $\sigma_{0}^{2}$, $\sigma_{n}^{2}$ and $\sigma_{0n}$ their variancies and covariance respectively and $c_{1}=0.01\max\left\{i\left(k_{max}\right)\right\},\;c_{2}=0.03\max\left\{i\left(k_{max}\right)\right\}$ two constants for avoiding possible arithmetic problems, and take their mean value, ie.,

(24) $$m_{SSIM}=\frac{1}{N}\sum_{n=1}^{N}SSIM\left(n\right).$$

We have run all the experiments on a 2.2 GHz Intel Core i7 processor and 16 GB RAM. Finally, in all the experiments we used $k_{max}=200$ and set $e_{max}=10^{-8}$. 

**Computational Complexity:** In groupwise alignment techniques computational complexity is a critical aspect, since they handle large sets of images. Considering the alignment process of a set of *N* images, of size $N_{x}\times N_{y}$ each, aiming to estimate a deformation vector with $N_{p}$ parameters, the computational complexity of LKE [24], LS centroid [25] and the proposed are presented in Table 1

where M=10 is the proposed number of clusters computed in the preprocessing stage of LKE, while 10<K<20 is the cost of computing the quantities necessary for the update of $\Delta p_{n}\left(k\right)$ in each iteration of LS centroid.

### 4.1. Experiment 1

**Figure 4:**
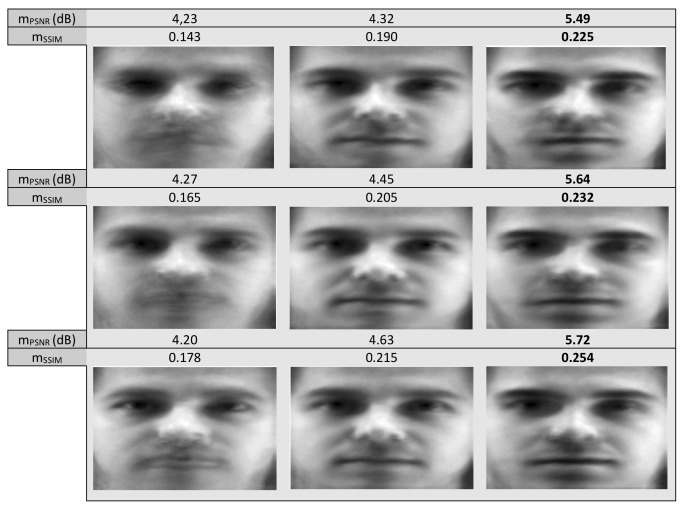
Mean misalignment images (first column) of 110 photometrically distorted images from Yale database and mean images resulting from their groupwise alignment by the LKE (second column) and the proposed technique (third column) with $\sigma^{2}=2$ (bottom line), $\sigma^{2}=6$ (middle line) and $\sigma^{2}=10$ (top line).

**Figure 5:**
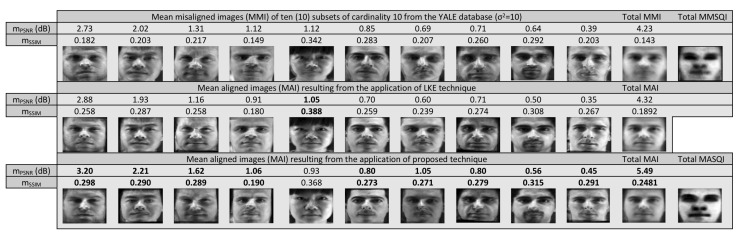
Mean Misalignment images (first row) of 110 photometrically distorted images ($\sigma^{2}=10$), distributed into 10 sets from Yale database. The mean images from each group (10 first comumns) the total mean image (11th column) and the total mean SQ images (last column), resulting from their groupwise alignment by the Lucas–Kanade Entropy (LKE) (second row) and the proposed technique (third row).

In this experiment, we are going to apply the proposed technique in solving the groupwise alignment problem in a set of geometrically and strongly photometrically distorted images of size 85×100 from the YALE database [28]. In order to evaluate our technique, we compare its performance against the Lucas–Kanade entropy (LKE)-based method [24] which is considered, although more computationally expensive than the proposed one, as a state of the art technique for solving groupwise and multi-modal image alignment problems. In Figure 4 the obtained results from the application of the methods in a set of 110 photometrically distorted images for three different values of the distortion parameter $\sigma^{2}$ are depicted with the corresponding $m_{PSNR}$ as well as $m_{SSIM}$ achieved by the methods after 200 iterations. It is clear that all methods succeed to visually improve the mean misalignment image although it is not clearly reflected the $m_{PSNR}$ as well as $m_{SSIM}$ values. It also seems that the performances of the two techniques are similar but with the computational cost of LKE much higher. More specifically, the computational cost per iteration for the proposed technique was 0.5 s while the cost of LKE was 1.1 s excluding the cost that is needed in its pre-processing step. However, as we can see in Figure 5 it is not true. Indeed, although the resulting total mean images seem to be, at least visually, alike, the mean images resulting from our method of each one of the ten aforementioned subsets are better not only visually but also in terms of their corresponding $m_{PSNR}$ as well as $m_{SSIM}$. Indeed, in most subsets, the proposed technique, even marginally, has better performance.

### 4.2. Experiment 2

**Figure 6:**
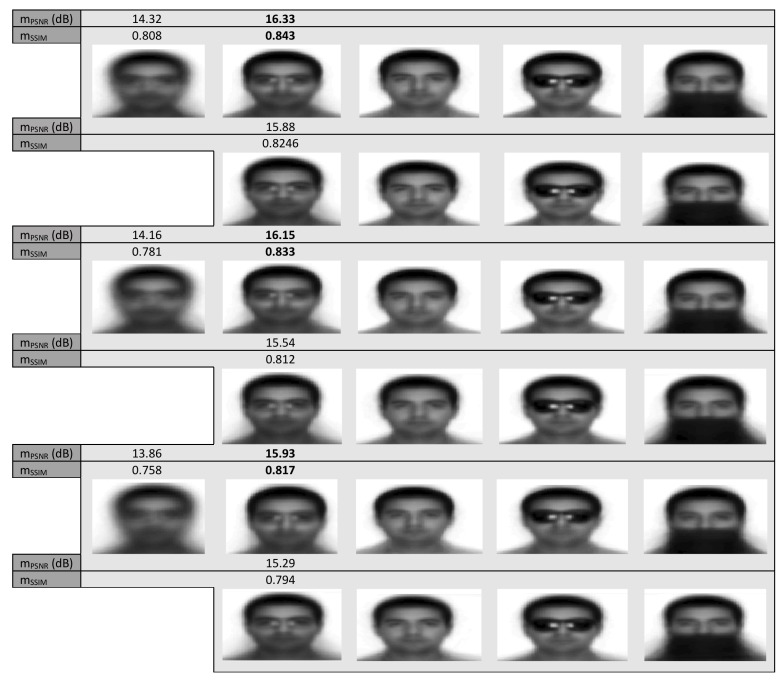
Mean misalignment images (first column for $\sigma^{2}=2$ (top), $\sigma^{2}=6$ (middle) and $\sigma^{2}=10$ (bottom)) of 300 images from AR database (100 neutral frontal pose (third column), 100 partially occluded by sunglasses (fourth column) and 100 partially occluded by scarfs (fifth column)) and mean images (second column) resulting from their groupwise alignment by the proposed first, third and fifth row and the LKE technique second, fourth and sixth row.

In this experiment, we are going to apply the methods on geometrically distorted images from the AR database [32]. Specifically, we used images of size 80×100 selected from the AR database. We tested the methods on a set containing 300 images composed by 100 in neutral frontal face pose, 100 partially occluded by sunglasses images and another subset of 100 more partially occluded by scarves images. Samples of these three different kinds of images are shown in the fourth column of Figure 2. Note that in this database the images are not centered on a common center of coordinates, meaning they are already geometrically distorted each other, occasionally including large rotation and/or translation distortions. The initial average of the warped images, the optimal ones as well as the optimal average of each one of the three aforementioned subsets obtained by the methods for three different values of the distortion parameter $\sigma^{2}$ are shown in Figure 6. Specifically, for the distortion parameter $\sigma^{2}$ taking the values 2, 6 and 10 respectively and as we are moving from the top to bottom of this figure, we can see in the odd rows the obtained results from the application of the proposed method while in the even ones the results from the application of LKE method. We can see that the performance of the proposed technique in terms of $m_{PSNR}$ as well as $m_{SSIM}$ is marginally better. The achieved mean alignment images per image subset are of high quality. In addition, the computational cost of the proposed technique is substantially lower. More specifically, the mean computational cost per iteration for the proposed technique was 1.00 s while the cost of LKE 2.05 s, leaving out the heavy cost that is needed in its pre-processing stage.

### 4.3. Experiment 3

**Figure 7:**
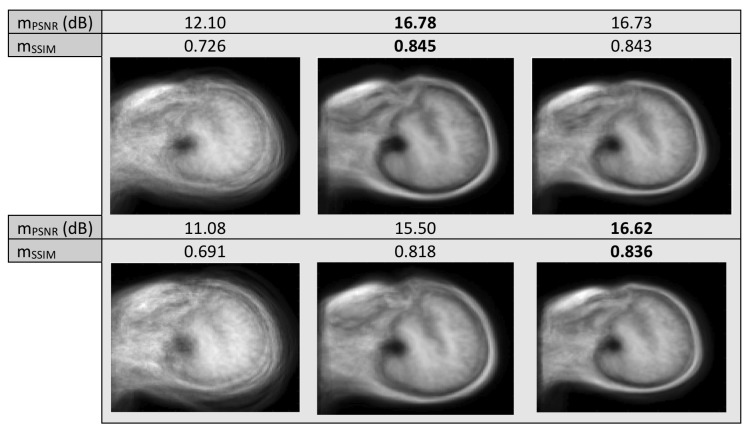
Mean misalignment images of MR images of the same modality with no warp (first row) and with warp of σ=5 (second row). Images before alignment (first column) and after alignment with LKE (second column) and the proposed (third column).

In this experiment we tested the methods with artificial MRI data obtained from Brainweb Database (https://www.mcgill.ca/bic/software/brainweb-mri-simulator) and real data from IXI Dataset (https://brain-development.org/ixi-dataset/), to test the alignment of images on the same or different modalities. We also applied artificial warps to the images, specifically small of size $\sigma^{2}=1$ and larger of size $\sigma^{2}=5$. We conducted a series of experiments with different MR image modalities, namely T1, T2, PD and MRA. First, we aim to align images from IXI Dataset containing the same slice, of the same modality (that is T1), from 100 different subjects of size 124×124. In Figure 7 we can see the “mean” image resulting after alignment, with artificial warping of $\sigma^{2}=5$ and no warping.

**Figure 8:**
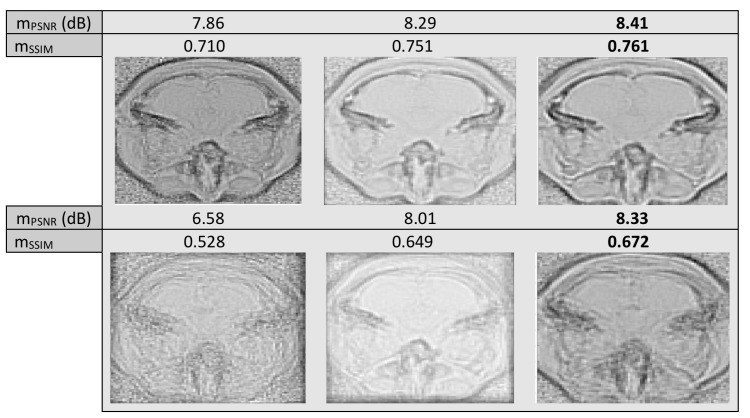
Mean misalignment images of T1, T2 MR images with warp of σ=1 (first row) and σ=5 (second row). Images before alignment (first column) and after alignment with LKE (second column) and the proposed (third column).

Next, we tested the methods with artificial data from the Brainweb Database, where we applied warps of $\sigma^{2}=1,5$ to the images. We used neighboring slices of T1, T2 and PD imaging, in a total of 35 images of size 109×91. In Figure 8 we can see the resulting “mean” image of LKE and the proposed method, as well as the original “mean” of the warped images.

**Figure 9:**
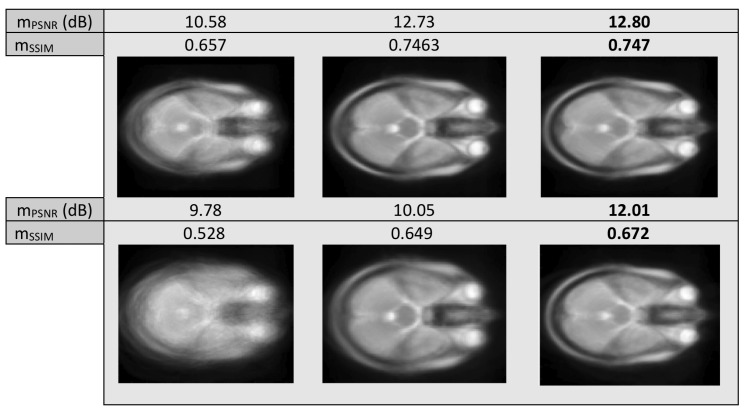
Mean misalignment images of T2, PD and MRA images with warp of σ=1 (first row) and σ=5 (second row). Image before alignment (first column) and after alignment with LKE (second column) and the proposed (third column).

Last, we tested using a dataset from IXI Dataset, aligning across the same slice different modalities T2, MRA and PD of size 128×128. In Figure 9 we may see the “mean” image for randomly selected slices from 28 subjects from each modality.

It is evident in all three experiments conducted with MR images, unimodal or multimodal, that the mean image resulting from the proposed method has more defined edges, especially when larger warps are applied to the images, leading to a more blurred original mean, even when in some cases, in terms of $m_{PSNR}$, as well as $m_{SSIM}$, the results of the two methods, are very close. The computational cost per iteration was 0.3,0.1 and 0.5 s, per experiment, for the proposed and 2.0,1.5 and 6.3 s, per experiment, for LKE, excluding preprocessing costs. As results indicate, clearly, iteration running time depends on the size of the image set. For LKE the increase in time cost may result in large overall time cost in the case of very large sets, i.e., over 1000 images, while in the case of the proposed the increase in iteration running time is very much smaller, allowing possible testing on sets of much larger size.

## 5. Conclusions

In this work, a new least-squares-based groupwise image registration method based on the use of self quotient images was proposed. The proposed technique has a very low computational cost. This was achieved by optimally defining a sequence of images whose limit was the desired but unknown “mean” image for solving the groupwise problem. Since the proposed technique is based on the self quotient images it was successfully used in solving the alignment problem of strongly photometrically distorted images, partially occluded images as well as in successfully solving the groupwise registration of multimodal MR images. Using as figures of merit the mean Peak Signal to Noise Ratio and mean Structural Similarity, the performance of the proposed technique from its application on a series of experiments was very good. The extensive evaluation of its performance against another state of the art groupwise registration techniques and its extension for solving the corresponding groupwise volume problem are currently under investigation. 

## Figures and Tables

**Table 1:** Computational complexity of groupwise alignment techniques.

LKE [24]	LS Centroid [25]	Proposed
$O(MN\left(N_{x}\times N_{y}\right)N_{p})$	$O(KN\left(N_{x}\times N_{y}\right)N_{p})$	$O(N\left(N_{x}\times N_{y}\right)N_{p})$,
